# Tuberculous prostatitis mimicking metastatic prostate cancer

**DOI:** 10.1590/0037-8682-0726-2021

**Published:** 2022-04-08

**Authors:** Serdar Aslan, Uluhan Eryuruk, Burhan Ozdemir

**Affiliations:** 1 Giresun University, Faculty of Medicine, Department of Radiology, Giresun, Turkey.; 2 Giresun Prof. A. Ilhan Ozdemir State Hospital, Urology Clinic, Giresun, Turkey.

**FIGURE 1:**
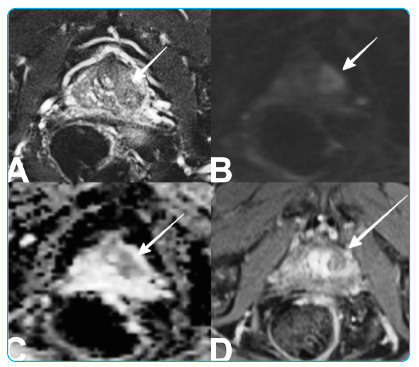
**A.** Axial T2-weighted images showed a focal and ill-defined low-signal intensity lesion in the left anterolateral peripheral zone close to the base (arrow). **B-C.** Diffusion-weighted images and apparent diffusion coefficient maps show the diffusion restriction of the lesion (arrow). **D.** Axial contrast-enhanced images show an early and prolonged ring enhancement of the lesion (arrow).

**FIGURE 2:**
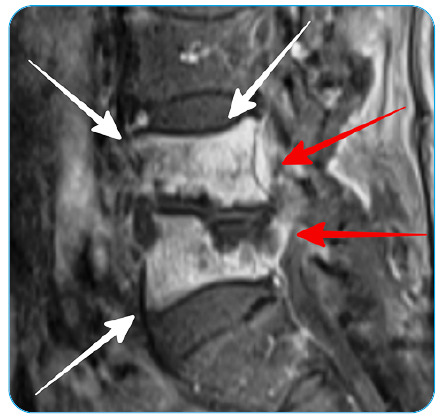
Sagittal contrast-enhanced images show diffuse enhancement of the L4 and L5 vertebral bodies (white arrows) and the accompanying soft tissues (red arrows).

An 80-year-old male presented with an obstructive lower urinary tract and intermittent low back pain lasting approximately 6 months. Digital rectal examination (DRE) revealed asymmetric prostate enlargement. Laboratory examination revealed elevated PSA, CRP, and sedimentation. Multiparametric magnetic resonance imaging (mp-MRI) and lumbar MRI were performed due to suspicion of prostate cancer and bone metastasis. On T2-weighted images (T2-WI), a low-signal intensity lesion with unclear borders was observed in the left anterolateral peripheral zone (Figure 1A). The defined lesion showed restricted diffusion and early ring enhancement (Figure 1B-D). Lumbar MRI revealed diffuse enhancement of the L4-L5 vertebrae, accompanied by soft tissue, which was observed (Figure 2). Prostate cancer and bone metastasis were considered in favor based on MRI findings, and a transrectal biopsy was performed. No neoplasia was detected on histopathological examination, and follicles with caseous necrosis and giant cells typical of tuberculous prostatitis were observed. The lesions observed in the vertebrae were evaluated in favor of Pott's abscess.

Isolated tuberculous prostatitis and concomitant Pott's abscess are extremely rare entity^1^. In DRE, prostate enlargement and hardened prostate areas are often detected, which is impossible to distinguish from prostate cancer^2^. Due to its relative rarity, MRI features have not been extensively described. Early ring enhancement in contrast-enhanced images is one of the most important imaging features defined in tuberculous prostatitis^3^. In conclusion, although tuberculous prostatitis can be diagnosed after histopathological examination, the characteristics of mp-MRI can guide the diagnosis.
